# Optimized steam boiler for soil steam disinfection: Structural design, CFD simulation, and field application

**DOI:** 10.1371/journal.pone.0340080

**Published:** 2025-12-26

**Authors:** Sipu Pan

**Affiliations:** College of Transportation Engineering, Nanjing Vocational University of Industry Technology, Nanjing, Jiangsu, China; University of Shanghai for Science and Technology, CHINA

## Abstract

Soil steam disinfection offers a chemical-free solution to soil-borne pathogens and continuous cropping obstacles; however, its adoption in facility agriculture is severely limited by the lack of mobile and efficient steam generation systems. Conventional electric or coal-powered boilers are too bulky and immobile for practical field application. To bridge this technological gap, this study developed an optimized gasoline-powered steam boiler utilizing an innovative Helmholtz-type pulse combustion system. Key components, including a dual-carburetor Y-shaped throat and a helical tailpipe configuration, were designed to enhance compactness and thermal efficiency—achieving a 67.11% reduction in length and 204% increase in heat transfer area compared to a straight pipe equivalent. Computational fluid dynamics simulations systematically optimized the spatial arrangement of four pulse combustors, revealing layout as a critical performance factor. The optimal configuration reduced water heating time to 407.1 s, 15.52 s faster than the average across 12 designs. Field validation demonstrated that a double-layer steam injection needle significantly improved thermal retention, maintaining soil temperatures ≥80°C for 63.84 minutes—27.02% longer than a single-layer design following 6 minutes of steam injection. These findings confirm the system’s efficacy in delivering lethal thermal dosage to soil pathogens. This work not only provides a practical mobile soil steam disinfection solution but also advances fundamental insights into the design of efficient thermal fluid systems for agricultural applications.

## 1. Introduction

The intensification of global agriculture through monoculture practices presents a critical paradox: while designed to maximize short-term productivity, it inadvertently undermines the foundational health of the soil ecosystem. This approach creates a vicious cycle, fostering the accumulation of soil-borne pathogens (including fungi, nematodes, and bacteria) and leading to the severe decline syndrome known as continuous cropping obstacles (CCO) [1–4]. CCO is not merely a agronomic challenge but a fundamental disruption of soil homeostasis, characterized by degraded soil structure, a dysbiotic microbial community, and the accumulation of allelopathic compounds [5–8]. Consequently, this results in stunted plant growth, substantial yield losses, and a major threat to both agricultural sustainability and global food security [9,10].

Conventional management strategies have struggled to resolve this paradox. Historically, reliance on broad-spectrum chemical fumigants, particularly methyl bromide, offered effective control but at an intolerable environmental cost, leading to its phase-out under the Montreal Protocol [11,12]. While alternative chemical agents exist, they universally present significant trade-offs among efficacy, specificity, and environmental safety, often causing detrimental effects on non-target soil biota and ecosystem health [13]. This has spurred intense interest in non-chemical, physical methods. Techniques such as soil solarization and hot water disinfection are constrained by prolonged treatment times, climatic dependencies, and inadequate soil penetration depth [14–17]. Flame disinfection, while chemical-free, often causes uneven heating, soil degradation, and high energy demands, limiting its practical efficacy and scalability [18–20].

Among these alternatives, soil steam disinfection (SSD) emerges as a theoretically ideal solution. By transferring latent heat from high-temperature steam (70–110°C) to the soil matrix, SSD effectively inactivates a broad spectrum of pathogens, weeds, and pests without leaving chemical residues [21,22]. Furthermore, it can improve soil structure and allows for immediate planting post-treatment [23,24]. However, a critical and often overlooked bottleneck prevents SSD from fulfilling its potential: the inherent limitation of its steam source. Conventional SSD systems rely on bulky, immobile electric or coal-fired boilers. These systems are fundamentally incompatible with the spatial constraints of facility agriculture and fail to meet the growing demand for sustainable, mobile, and efficient field operations. The central problem thus shifts from the principle of thermal disinfection to the engineering of its delivery, specifically the challenge of generating adequate steam with sufficient thermal efficiency within a compact, mobile, and environmentally sound platform.

This study addresses this fundamental engineering challenge by introducing a novel steam generation system that reimagines the core energy conversion process. It moves beyond incremental improvements to propose a paradigm shift: a compact, gasoline-powered boiler utilizing pulsed combustion technology. Unlike steady-state combustion, pulsed combustion in Helmholtz-type combustors achieves exceptionally high rates of heat transfer and thermal efficiency [25,26] The system integrates an array of four such optimized combustors, directly submerged in water, to maximize heat exchange surface area and minimize steam generation latency. The originality of this work is twofold: First, it presents the first application of a multi-unit pulsed combustion system specifically designed for mobile soil disinfection, overcoming the energy-mobility dilemma. Second, it systematically investigates—through Computational Fluid Dynamics (CFD) simulation and field validation—the critical yet unexplored relationship between the spatial configuration of multiple heat sources and the overall thermal dynamics of the system. This research not only delivers a practical solution for sustainable agriculture but also provides new insights into the design principles of high-efficiency, mobile thermal energy systems.

## 2. Methods

### 2.1. Structure design of the helmholtz-type pulse combustor

#### 2.1.1. Structural composition.

Pulse combustion, as a distinctive unsteady combustion mode, exhibits periodic variations in characteristic state parameters (e.g., combustion chamber pressure, temperature, and exhaust flow velocity) throughout its operation. The sustainability of pulse combustion fundamentally depends on the phase relationship between heat release dynamics and gas pressure oscillations. Specifically, the oscillatory process becomes self-sustaining and amplified when heat addition occurs during maximum gas compression or when heat extraction coincides with maximum expansion. This phase synchronization mechanism represents a critical feature distinguishing pulse combustion from conventional steady-state combustion systems.

**Fig 1:**
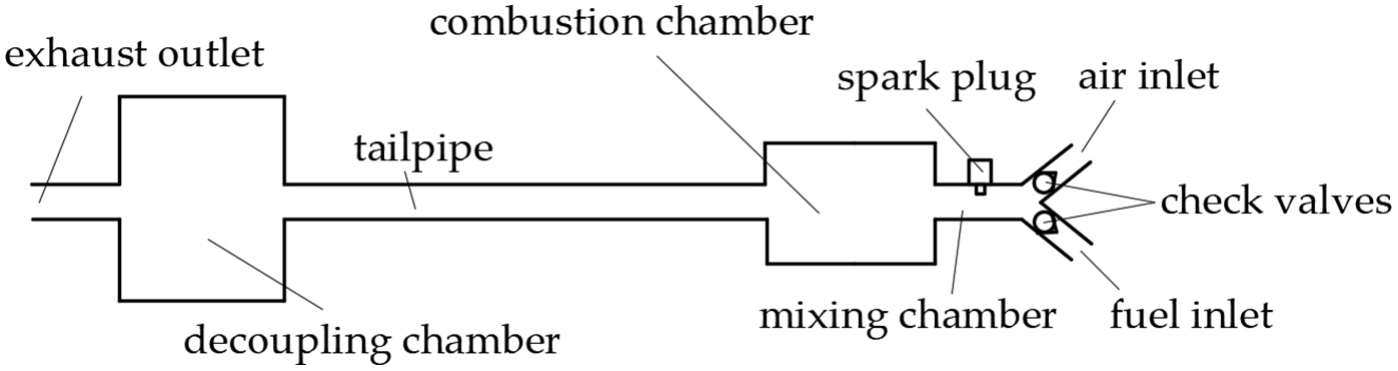
Structure of Helmholtz-type pulse combustor.

The Helmholtz-type pulse combustor, as illustrated in Fig 1, comprises several key structural components: an air inlet, fuel inlet, spark plug, mixing chamber, combustion chamber, tailpipe, decoupling chamber, and exhaust outlet. Both the air and fuel inlets incorporate check valves to ensure unidirectional flow into the mixing chamber. A spark plug mounted on the mixing chamber wall initiates combustion of the air-fuel mixture. Subsequent vigorous combustion occurs within the combustion chamber, generating substantial thermal energy. Exhaust gases then flow sequentially through the tailpipe and into the decoupling chamber before exiting through the exhaust outlet, during which process residual heat continues to be released. The decoupling chamber features a substantially large internal volume that serves multiple important functions: it provides effective acoustic damping to reduce noise emissions while simultaneously maintaining the required acoustic boundary conditions at the tailpipe exit plane. This dual functionality is crucial for preserving the combustor’s characteristic acoustic properties that enable sustained pulse combustion operation.

#### 2.1.2. Working cycle.

**Fig 2:**
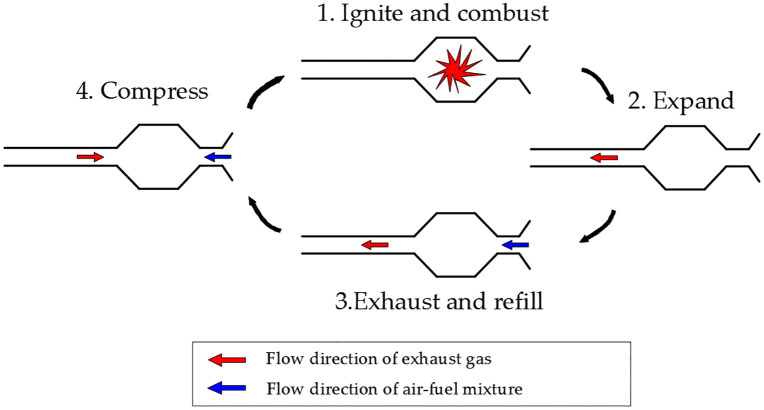
Working cycle of Helmholtz-type pulse combustor.

The working cycle of a Helmholtz-type pulse combustor can be divided into four distinct phases, as illustrated in Fig 2.

The first phase involves ignition and combustion, where fuel and air enter the mixing chamber through their respective check valves. The spark plug generates an electrical discharge to ignite the mixture, which then flows into the combustion chamber due to inertial forces. The subsequent rapid combustion and heat release cause a pressure increase that forces combustion products to flow toward both the tailpipe and mixing chamber. When the mixing chamber pressure exceeds the supply pressures of both fuel and air, the check valves close sequentially.

During the expansion stage, combustion is almost over while the exhaust gases continue moving outward due to inertia. With both check valves closed, the combustion products can only exit through the tailpipe, causing a gradual pressure decrease in the combustion chamber until a partial vacuum forms.

During the exhaust and refill phase, the combustion exhaust continues moving toward the tailpipe under inertial forces while the combustion chamber pressure keeps decreasing. When the mixing chamber pressure drops below the supply pressures of both fuel and air, the resulting pressure differential causes the check valves at the respective inlets to reopen, allowing fresh air and fuel to refill the mixing chamber. The newly formed combustible mixture then enters the combustion chamber following the residual exhaust gases from the mixing chamber. Meanwhile, due to persistent inertial effects, the exhaust gases flow within the tailpipe maintains its outward motion while gradually decelerating, requiring an extended duration to achieve complete flow reversal.

The final compression phase occurs as the exhaust gas velocity in the tailpipe decreases to zero and reverses direction under the previous vacuum effect. The returning exhaust gases compress together with the fresh charge in the combustion chamber, causing a rapid pressure rise. The resulting high temperature and pressure conditions auto-ignite the fresh mixture, initiating the next cycle without requiring spark plug intervention.

It is noteworthy that the spark plug operates exclusively during the initial startup phase of the pulse combustor. Once stable operation is achieved, the system maintains self-sustained combustion without requiring spark-induced ignition of the air-fuel mixture. This auto-ignition capability, enabled by the thermal compression of fresh charge during the compression phase, represents a distinctive characteristic of Helmholtz-type pulse combustors that differentiates them from conventional combustion systems.

### 2.2. Structure design of the steam boiler

#### 2.2.1. Structural composition.

The steam boiler, as the steam source for SSD, constitutes a critical component of the disinfection system, whose operational reliability determines the successful implementation of disinfection procedures. Accordingly, the following structural and functional requirements must be satisfied:

(1) The steam boiler shall feature a compact design with minimized dimensions for enhanced mobility and compatibility with tracked chassis mounting.
(2) Functioning as a pressure vessel under high-temperature and high-pressure conditions, all components must demonstrate adequate mechanical strength to withstand design pressures, ensure leak-proof connections at all joints, and exhibit sufficient corrosion resistance, while maintaining operational simplicity and serviceability.
(3) When employing pulse combustors as heat sources, the system must guarantee reliable ignition and maintain stable operation without combustion interruptions or unexpected shutdowns.
(4) The pulse combustors must provide adjustable power output to ensure the steam boiler’s production capacity meets disinfection demands.

**Fig 3:**
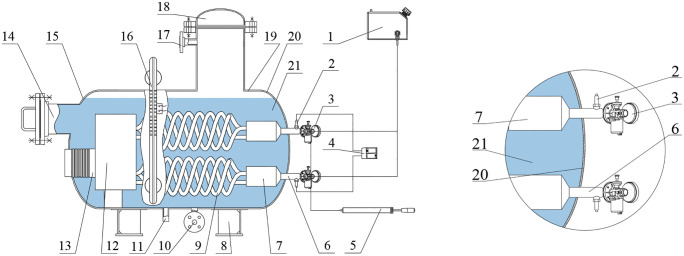
Structure of the steam boiler. (a) Overall structure; (b) Detailed structure. 1. fuel tank 2. spark plug 3. carburetor 4. high-voltage generator 5. air pump 6. throat 7. combustion chamber 8. base frame 9. tailpipe 10. water inlet 11. drainage port 12. decoupling chamber 13. bellows 14. maintenance hatch 15. left end cap 16. water level gauge 17. steam outlet 18. steam dome 19. tank body 20. right end cap 21. water.

In accordance with the structural and functional requirements for steam boilers, this study developed a steam boiler utilizing pulse combustors as the heat source, with its configuration illustrated in Fig 3(a). The boiler adopts a horizontal layout, offering advantages of low center of gravity for enhanced mobility and stability against tipping. The system comprises two primary components: the pulse combustor components and the cylinder components.

The horizontally oriented pulse combustor components consist of a fuel tank, spark plug, carburetor, high-voltage generator, air pump, throat, combustion chamber, tailpipe, decoupling chamber, and bellows. The cylinder components include a base frame, water inlet, drainage port, maintenance hatch, left end cap, water level gauge, steam outlet, steam dome, tank body, and right end cap. The air pump is responsible for initiating the pulse combustor’s startup sequence. Note that ports for installing pressure gauges, thermometers, and safety valves are not shown in Fig 3(a).

The left end of the pulse combustor is welded to the left end cap through a bellows connection, which not only ensures smooth exhaust gas flow but also compensates for dimensional changes caused by thermal expansion and contraction of the pulse combustor during operation. The right end of the pulse combustor is welded to the right end cap via the throat, effectively isolating both the spark plug and carburetor from the cylinder components, with detailed configuration shown in Fig 3(b). The tank body contains water that completely submerges key components of the pulse combustor, including the combustion chamber, tailpipe, and decoupling chamber.

#### 2.2.2. Analysis of heat transfer pathway and modes.

**Fig 4:**
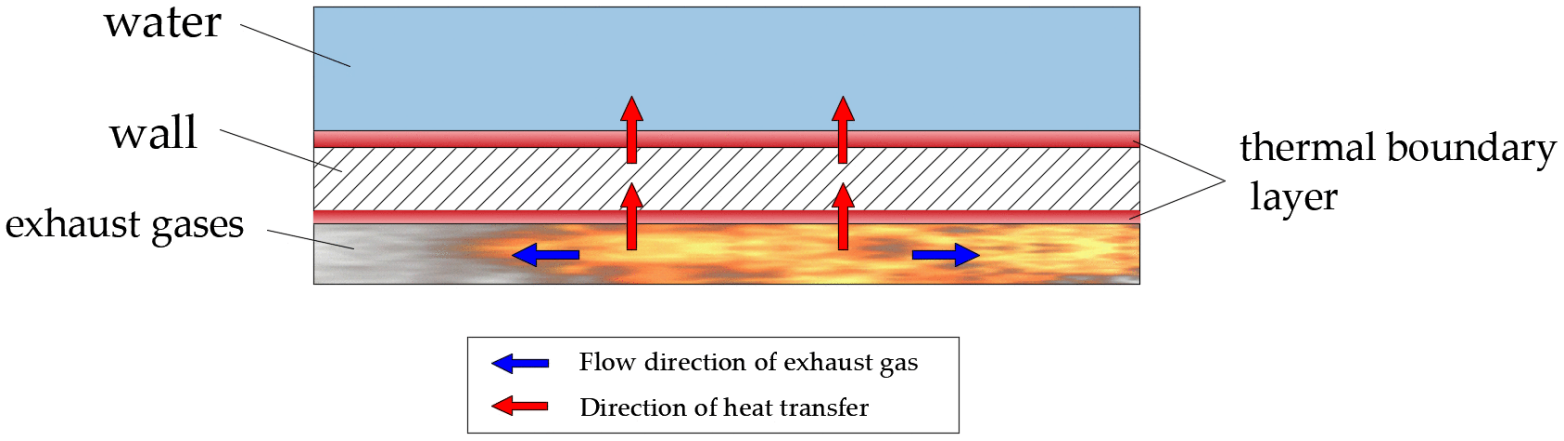
Heat transfer pathway.

**Fig 5:**
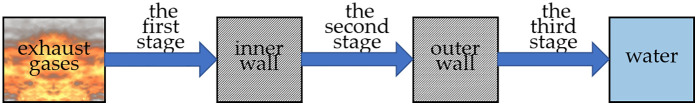
Three stages of heat transfer.

The heat transfer pathway of exhaust gases is illustrated in Fig 4. It should be noted that the term “wall” in Fig 4 collectively refers to the structural boundaries of the throat, combustion chamber, tailpipe, decoupling chamber, and bellows. The surface adjacent to exhaust gases is designated as the inner wall, while the water-facing surface is identified as the outer wall. The complete heat transfer process from exhaust gases to water occurs through three distinct stages, as detailed in Fig 5.

In the first stage, heat is transferred from the high-temperature exhaust gases to the inner wall surface through a combination of convective heat transfer and thermal radiation. The convective heat transfer itself consists of two mechanisms: conduction within the thin thermal boundary layer that forms adjacent to the cooler inner wall surface, and convection in the fully mixed flow region beyond this boundary layer. Simultaneously, thermal radiation transfers heat omnidirectionally to the inner wall. This stage is primarily characterized by single-phase exhaust gases heat transfer, although minor condensation heat transfer occurs when trace amounts of water vapor in the exhaust gases condense on subcooled inner wall surfaces below the saturation temperature, releasing latent heat through phase change.

The second stage involves conductive heat transfer through the wall structure from the inner to outer surfaces. This process can be approximated as a one-dimensional steady-state conduction problem, with the heat transfer rate determined by the thermal conductivity of the wall material, the wall thickness, the surface area, and the temperature gradient between the inner and outer walls.

In the third stage, convective heat transfer dominates at the interface between the outer wall and the water. Similar to the first stage, a thermal boundary layer forms near the outer wall surface, with conduction dominating within the boundary layer and convection in the bulk fluid.

The convective heat transfer process in the third stage can be further categorized into three distinct phases. During the initial operation of the pulse combustor, the high thermal load combined with relatively low outer wall superheating causes gradual temperature increase in the thermal boundary layer adjacent to the outer wall surface, though the water temperature remains below the saturation point at the corresponding pressure. This non-uniform temperature distribution establishes a density gradient, generating buoyancy-driven flow where warmer boundary layer water rises while cooler surrounding water descends, establishing natural convection within the confined space. As system operation continues, the bulk water temperature progressively increases. When sufficient outer wall superheat develops, the boundary layer water reaches saturation temperature, initiating phase change and bubble formation. Further superheat enhancement promotes bubble detachment and buoyant rise through the water column, significantly intensifying heat transfer. This regime represents subcooled boiling, as the bulk water temperature remains below saturation conditions. Eventually, when the bulk water attains saturation temperature, the heat transfer mechanism transitions from subcooled boiling to stable film boiling, initiating continuous saturated steam generation.

A critical distinction exists between the convective mechanisms in the first and third stages. The initial stage features forced convection heat transfer driven by exhaust gas pressure differentials, while the terminal stage exhibits free convection heat transfer arising from thermally-induced density gradients under gravitational influence. This fundamental difference in driving potential necessitates separate heat transfer correlations for accurate thermal analysis of each regime.

### 2.3. Main components design

#### 2.3.1. Throat.

Based on previous research findings, meeting the required steam output would necessitate at least eight pulse combustors to satisfy power demands, which would result in an excessively large and structurally complex steam boilers unit that contradicts the compact design requirements established earlier. To resolve this issue while maintaining the necessary power output, this study developed an innovative dual-carburetor pulse combustor design where each unit incorporates two carburetors operating simultaneously. This configuration doubles both the fuel and air intake and power output per combustor while reducing the total number of required units by half, which means only four combustors are needed to achieve the target power.

**Fig 6:**
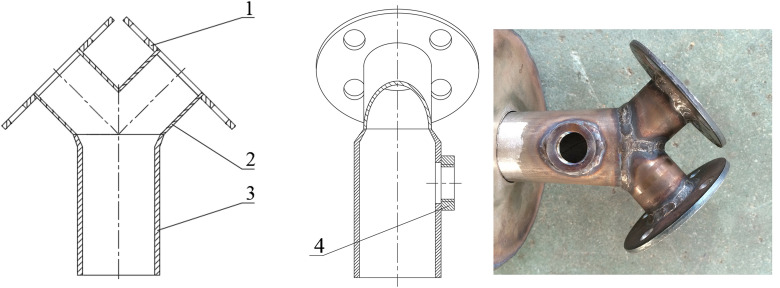
Structure of the throat. (a) Front cutaway view; (b) Left cutaway view; (c) Photo. 1. connection flange 2. branch pipe 3. main pipe 4. spark plug mounting base.

To achieve the target power output while minimizing the number of combustor units—a critical requirement for system compactness—an innovative dual-carburetor design was adopted for each pulse combustor. This approach necessitated a fundamental redesign of the throat section. The conventional single-inlet throat was replaced with a Y-shaped configuration (Fig 6) to efficiently merge two fuel-air streams from the carburetors into a single flow entering the combustion chamber. This design served a dual purpose: it acted as a mixing chamber to ensure a homogeneous mixture prior to combustion, while simultaneously achieving a significant reduction in overall system size and complexity. The diameter of the spark plug mounting base (16 mm) was determined to ensure reliable ignition while minimizing flow disruption.

#### 2.3.2. combustion chamber.

The combustion chamber design was optimized with the primary objective of maximizing volumetric heat release rate while ensuring structural integrity and flame stability. The key design variables were the chamber diameter and length, with their ratio constrained to a conventional range of 1.5–2.5 to maintain efficient combustion dynamics. The optimal dimensions were determined by solving Equations (1) and (2) iteratively to meet the target power output while satisfying the geometric constraint:


$$q_{r}=\frac{P_{r}}{V_{r}}$$ (1)

$$V_{r}=\frac{\text{1}}{\text{4}}\pi D_{r}^{2}\cdot L_{r}$$ (2)

where *q*_*r*_ is the volumetric heat release rate of the combustion chamber in kW/m^3^; *P*_*r*_ is the power output of the pulse combustor in kW; *V*_*r*_ corresponds to the combustion chamber volume in m^3^; *D*_*r*_ indicates the internal diameter of the combustion chamber in m; and *L*_*r*_ signifies the length of the combustion chamber in m. The meanings of the symbols used in this article are provided in the S1 Appendix, and the same applies hereafter.

The length-to-diameter ratio was initially set at 2:1 (*L*_*r*_ = 2 *D*_*r*_). Based on these calculations, the chamber diameter *D*_*r*_ was determined to be 0.08 m, resulting in a chamber length *L*_*r*_ of 0.16 m. A smaller diameter would risk excessive *q*_*r*_ leading to instability, while a larger diameter would reduce *q*_*r*_ below efficient operation levels. Thus, the selected dimensions represent a balance between high power density and operational reliability.

#### 2.3.3. Tailpipe.

The exhaust gases generated within the combustion chamber are propelled into the tailpipe under pressure, maintaining elevated temperatures. The high-velocity pulsating exhaust gases flow effectively disrupts the thermal boundary layer, thereby enhancing convective heat transfer efficiency and establishing the tailpipe as one of the primary heat exchange surfaces in the steam boiler.

The geometric parameters of the tailpipe – including its length, cross-sectional area, and configuration – critically influence both the ignition characteristics and operational stability of the pulse combustor, while simultaneously determining the thermal efficiency of the steam boiler. The pulse combustor requires a minimum tailpipe length to meet operational conditions – below this critical threshold, ignition becomes impossible. While increasing tailpipe length enhances heat transfer surface area and improves thermal efficiency, it simultaneously elevates flow resistance losses. These increased losses adversely affect the combustor’s self-aspiration capability and operational stability. Consequently, optimal tailpipe dimensions must be determined within a constrained design window that balances these competing factors.

The tailpipe cross-sectional area is calculated using Equation (3):


$$\sum_{i=1}^{n}S_{w(i)}\;\;:S_{r}=0.15\sim 0\mathrm{.3}$$ (3)

where *S*_*w(i)*_ is cross-sectional area of the i^th^ tailpipe in m^2^; *S*_*r*_ is combustion chamber cross-sectional area in m^2^.

Maintaining constant total tailpipe cross-section while increasing the number of tailpipes enhances heat transfer surface area, thereby improving thermal efficiency. This study employs three tailpipes, with individual inner diameters calculated via Equation (4):


$$3\times \frac{\text{1}}{\text{4}}\pi {D_{w}}^{2}=0.2\times \frac{\text{1}}{\text{4}}\pi {D_{r}}^{2}$$ (4)

where *D*_*w*_ is individual tailpipe inner diameter in m. Substituting into the calculation, *D*_*w*_ =0.02m.

**Fig 7:**
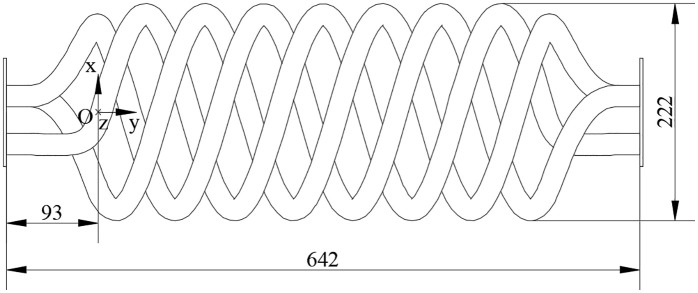
Structure diagram of tailpipe.


The tailpipe design involves a critical trade-off between functional requirements (maintaining combustion stability), heat transfer efficiency, and spatial constraints. While a longer tailpipe provides greater heat transfer area, it also increases flow resistance, potentially compromising the combustor’s self-aspiration capability and stability. To resolve this conflict, a helical coil configuration was adopted, fabricated from 06Cr19Ni10 stainless steel tubing (see Fig 7 for schematic). The three-dimensional coordinate system in Fig 7 originates at point O (93 mm from the tailpipe inlet), with the z-axis oriented inward. The left-handed helical coil follows the spatial Equation (5):

$$\left\{ \;\;\;\begin{array}{c} x=99*\cos(\theta *360*2.5)\\ z=99*\sin(\theta *360*2.5)\\ y=\theta *180*2.5 \end{array}\right.$$ (5)

where *θ* is coefficient (0≤*θ*≤1).This design strategy effectively decouples the functional length requirement (1952 mm, based on previous research) from the spatial footprint. The resulting helix, with a lead of 180 mm and an elevation angle of 16.1°, achieved the dual objectives of preserving the necessary acoustic length for stable operation while reducing the installed length by 67.11% and increasing the heat transfer area by 204% compared to an equivalent straight pipe, thereby optimizing both performance and compactness.

Alternative tailpipe configurations, including U-bend and multi-pass layouts, were initially considered. While the U-bend offered packaging advantages, it introduced unacceptable flow losses that compromised pulsation stability. The multi-pass layout provided superior heat transfer but presented prohibitive manufacturing complexity. The helical coil configuration was ultimately selected as it optimally balanced the competing criteria of hydraulic performance, heat transfer efficiency, manufacturability, and spatial footprint.

### 2.4. Structural layout optimization

#### 2.4.1. Determination of optimization parameters.

**Fig 8:**
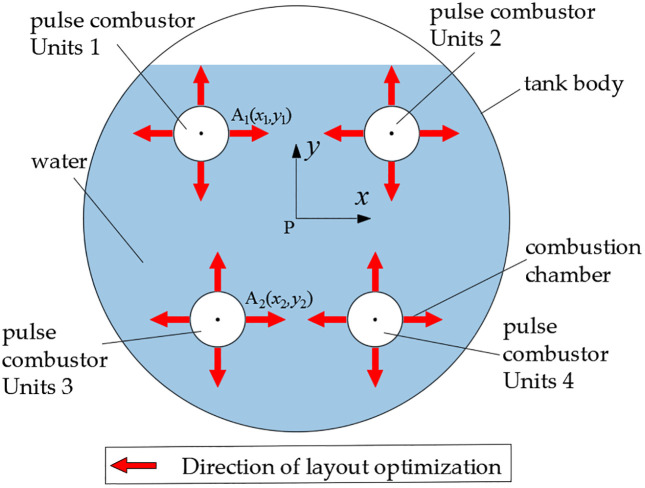
Optimal parameters.

As previously described, the designed steam boiler incorporates four pulse combustors. Following the determination of individual component dimensions for each pulse combustor, the remaining critical parameter requiring specification involves the spatial arrangement of these four units within the boiler structure. Given that the combustion chamber represents the primary site for fuel-air mixture combustion, this region maintains significantly higher temperatures compared to other system components. A cross-sectional plane through the central axis of the combustion chambers was established, yielding the sectional view presented in Fig 8. The four pulse combustors are designated as Units 1 through 4 for identification purposes. A Cartesian coordinate system was implemented with point P (the geometric center of the tank body) serving as the origin. Considering the boiler’s bilateral symmetry, only the x < 0 region was selected for analysis. The geometric centers of combustion chambers for Units 1 and 3 (denoted as points A_1_ and A_2_ respectively) possess coordinates (x_1_, y_1_) and (x_2_, y_2_). Consequently, the optimization parameters for the steam boiler design are reduced to these four coordinate variables: x_1_, y_1_, x_2_, and y_2_.

**Fig 9:**
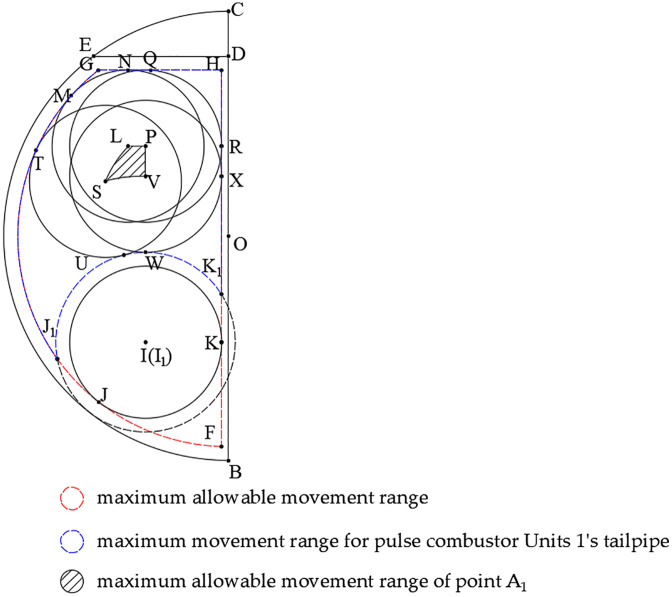
Optimal parameters range.

To ensure adequate steam storage capacity and prevent water intrusion into the steam outlet due to violent boiling, the maximum water level inside the boiler was set at 0.9 times the tank body diameter. As shown in Fig 9, line segment ED represents this maximum water level, with the dimensional relationship L_ED_ = 0.9L_BC_.

Fig 3(a) demonstrates that the outer diameter of the helical tailpipe exceeds that of the combustion chamber, necessitating the use of tailpipe outer dimensions for determining optimization parameter limits. Accounting for manufacturing tolerances and assembly clearances, a 20 mm safety margin was implemented, establishing minimum spacing requirements: (1) between tailpipes and maximum water level, (2) between tailpipes and tank body wall, and (3) between adjacent tailpipes. These constraints define the maximum allowable movement range, represented by the dashed area bounded by segments GH and HF and arc FG in Fig 9.

Building upon previous research indicating slower temperature rise in bottom water regions prolongs steam generation, pulse combustor Units 3 was positioned at the lowest feasible location (minimizing y_2_). Constructing circle I with tailpipe outer diameter tangent to arc FG and segment HF yields intersection points J and K, with point I representing the lowest possible position for combustion chamber center A_2_. Expanding this boundary, circle I_1_ was drawn with diameter increased by 40 mm (dashed circle in Fig 9), intersecting arc FG and segment HF at J_1_ and K_1_. The area enclosed by arcs GJ_1_/J_1_K_1_ and segments K_1_H/HG defines the maximum movement range for pulse combustor Units 1’s tailpipe.

The geometric constraints for the maximum allowable movement range of pulse combustor Units 1’s combustion chamber center (point A_1_) were determined through systematic construction as follows:

Constructing circle L with diameter equal to the tailpipe outer dimension, tangent to both arc GJ_1_ and line segment HG, yields intersection points M and N.

Similarly, circle P of identical diameter, tangent to segments K_1_H and HG, generates intersections at points R and Q.

Circle V, constructed with the same diameter while tangent to segment K_1_H and arc J_1_K_1_, produces intersection points X and W.

Circle S, tangent to arcs GJ_1_ and J_1_K_1_ with equivalent diameter, intersects at points T and U.

The final boundary was established through a series of geometric constructions: first, arc LS was created parallel to arc GJ_1_; then, arc SV was drawn parallel to arc J_1_K_1_; finally, points V to P and P to L were connected with straight segments, collectively forming the enclosed boundary.

The resultant shaded area, bounded by arcs LS/SV and connecting segments VP/PL, defines the optimized movement domain for positioning the combustion chamber center of pulse combustor Units 1 (A_1_). This geometrically-derived solution ensures proper component clearance while maximizing thermal performance through strategic placement within the constrained boiler volume.

**Fig 10:**
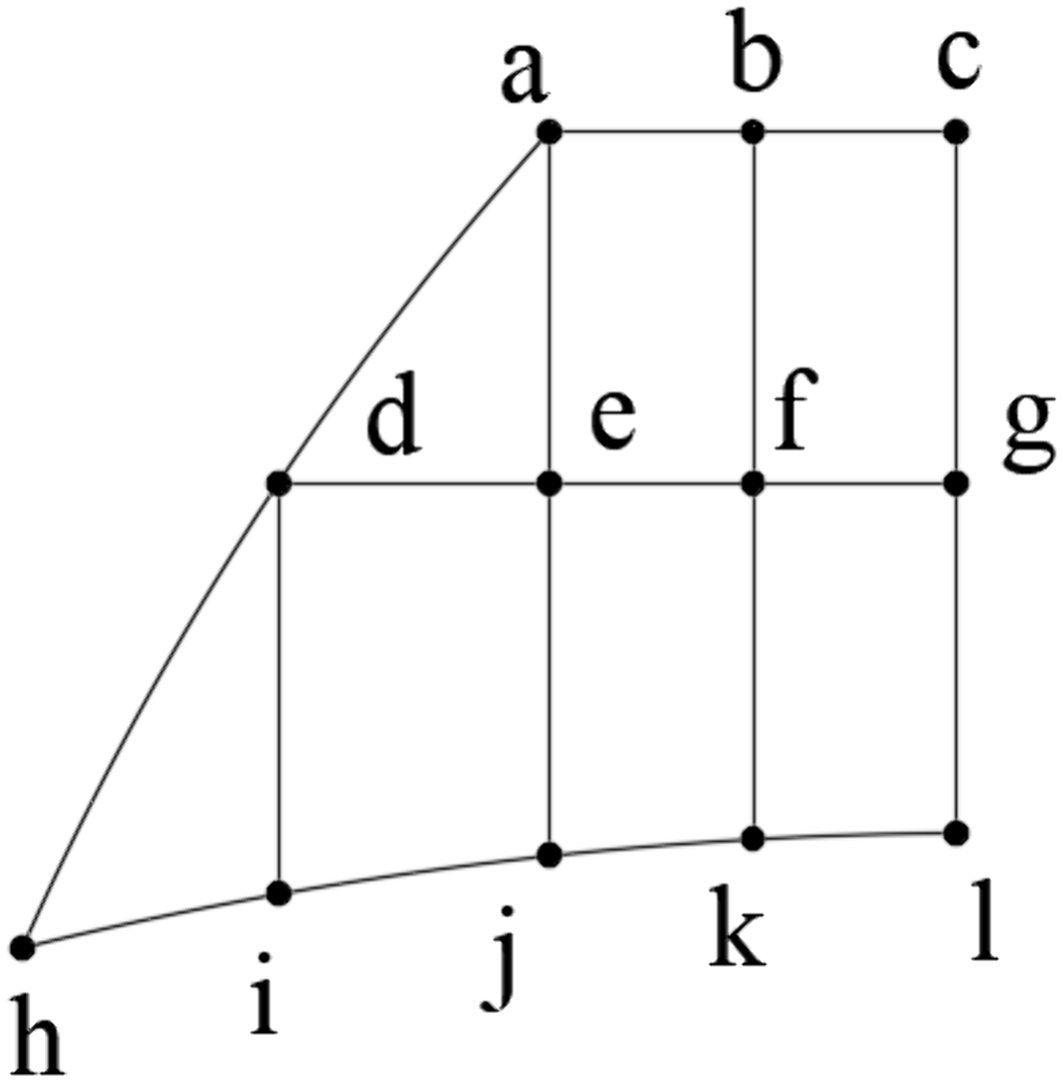
Mesh of optimal parameters range.

To determine the optimal position of point A_1_, the shaded region in Fig 9 was discretized into a grid pattern (Fig 10), generating 12 nodal points (a-l) with equal segment spacing (L_ab_ = L_bc_ and L_cg_ = L_gl_). The coordinates of these nodal points are provided in Table 1.

**Table 1:** Nodes coordinates.

Node	x_1_	y_1_	Node	x_1_	y_1_
a	−145.3	130	g	−120	108.1
b	−132.7	130	h	−178.2	79.1
c	−120	130	i	−162.3	82.5
d	−162.3	108.1	j	−145.3	85.0
e	−145.3	108.1	k	−132.7	86.0
f	−132.7	108.1	l	−120	86.3

#### 2.4.2. Numerical simulation equations.

Based on the Eulerian homogeneous flow model, the fluid domain is treated as a homogeneous mixture comprising liquid and vapor phases with their respective volume fractions, as described by the volume fraction Equation (6):


$$a_{l}+a_{g}=1$$ (6)

where *a*_*l*_ represents the liquid phase volume fraction in the fluid in %; *a*_*g*_ denotes the vapor phase volume fraction in the fluid in %.

The governing equations for the two-phase flow system include:

Continuity Equation (7):


$$\frac{\partial \rho_{m}}{\partial t^{\prime }}+div(\rho_{m}{\vec{v}}_{m})=0$$ (7)

where $\rho_{m}\;$ represents density of the two-phase mixture in kg/m³; *t’* denotes the time in s; ${\vec{v}}_{m}$ is velocity vector of the two-phase flow in m/s.

Momentum Equation (8):


$$\frac{\partial }{\partial t^{\prime }}(\rho_{m}{\vec{v}}_{m})+div(\rho_{m}{\vec{v}}_{m}{\vec{v}}_{m})=-divp+\rho_{m}\vec{g}+\vec{F}+div\left[\mu_{m}(div{\vec{v}}_{m}+div{{\vec{v}}^{T}}_{m})\right]\;\mathrm{\backslash vspace}\mathrm{-10mm}+\;div(\sum_{k=1}^{2}\varphi_{k}\rho_{k}{\vec{v}}_{dr,k}{\vec{v}}_{dr,k})$$ (8)

where *p* represents pressure in Pa; $\vec{g}$ is gravitational acceleration vector in m/s²; $\vec{F}$ is body force vector in N/kg; $\mu_{m}$ is dynamic viscosity of the two-phase flow in Pa·s; $\varphi_{k}$ is volume fraction of phase k in %; $\rho_{k}$ is density of phase k in kg/m³; ${\vec{v}}_{dr,k}$ is drift velocity vector of phase k in m/s.

Energy Equation (9):


$$\frac{\partial }{\partial t^{\prime }}\sum_{k=1}^{2}\varphi_{k}\rho_{k}h_{k}+div\sum_{k=1}^{2}\left[\varphi_{k}v_{k}(\rho_{k}h_{k}+p)\right]=div(\lambda_{eff}divT^{\prime })+S_{E}\mathrm{\backslash ]}$$ (9)

where *h*_*k*_ represents specific enthalpy of phase k in J/kg; *v*_*k*_ is velocity of phase k in m/s; $\lambda_{\text{eff}}$ is effective thermal conductivity in W/(m·K); *T’* is temperature in K; S_E_ is source term in J/kg.

Phase Change Relations Equations (10) and (11):


$$M_{l}=-\omega a_{l}\rho_{l}\frac{\Delta t}{t_{s}}$$ (10)

$$M_{g}=\omega a_{l}\rho_{l}\frac{\Delta t}{t_{s}}$$ (11)

where *M*_*l*_ represents liquid phase mass source term in kg/(m³·s); *ω* is relaxation factor in s ⁻ ¹; $\rho_{l}$ is liquid phase density in kg/m³; *M*_*g*_ is vapor phase mass source term in kg/(m³·s).

#### 2.4.3. Meshing and boundary condition setting.

This study optimized the spatial configuration of four pulse combustors by computationally analyzing the thermal evolution of the water inside the tank body. The computational domain was defined as the region bounded by arc BE and segments ED/BD in Fig 9, excluding the combustion chamber volumes of combustion chambers Units 1 and 3. The undetermined position of combustion chambers Units 1 generated 12 distinct domain configurations, each designated according to its corresponding nodal nomenclature (a-l).

**Fig 11:**
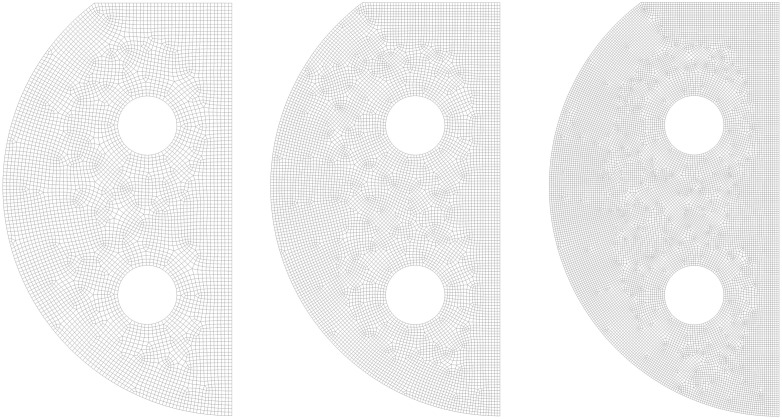
Three sets of meshes of Group l. (a) Coarse mesh; (b) Medium mesh; (c) Fine mesh.

To ensure the computational results were independent of mesh density, a mesh sensitivity analysis was first conducted, using Group l as a representative example. Three sets of meshes with increasing refinement—coarse, medium, and fine—were generated for the optimal configuration, which adopts quadrilateral mesh elements and the method of unstructured meshing. The results for the three meshes are shown in Fig 11. The volume-weighted average temperature of the water domain was monitored as the key parameter for assessment.

As illustrated in Fig 9, the boundary condition for arc BE is defined as “WALL”, while that for line segment ED is specified as “PRESSURE_OUTLET”, and “SYMMETRY” is assigned to line segment BD. When the pulse combustor’s main body is submerged in water, directly measuring the temperature rise process of the combustion chamber’s outer wall proves infeasible. Consequently, within the computational model, it is impossible to prescribe either a fixed temperature value or a temperature-rise equation to this surface.

Notably, from the standpoint of structural optimization, the optimal structural configuration should remain invariant regardless of variations in the combustion chamber’s outer wall’s temperature-rise characteristics. To enhance the model’s fidelity to real-world conditions, the boundary condition of the combustion chamber’s outer wall is set as “WALL”. The temperature-rise profile is based on findings from prior research [27]. This data is subsequently integrated into the model via the User-Defined Function (UDF) interface, ensuring seamless incorporation of custom physical behavior into the simulation framework.

**Fig 12:**
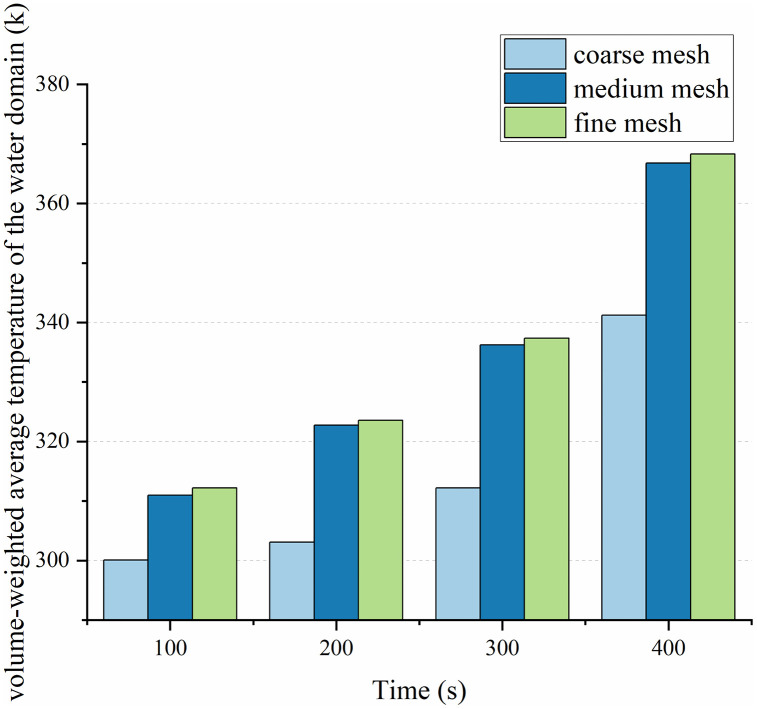
Results of the mesh sensitivity analysis: Evolution of the volume-weighted average temperature.

The results of the simulations for the three meshes at various time intervals are presented in Fig 12. A significant discrepancy is evident between the coarse mesh and the two finer meshes. For instance, at t = 400 s, the coarse mesh predicted a temperature of 341.20 K, which underestimates the fine mesh result (368.32 K) by approximately 27.12 K or 7.4%. This substantial deviation confirms that the coarse mesh is inadequate to resolve the complex heat transfer and phase change dynamics, introducing unacceptable numerical errors.

In stark contrast, the results from the medium and fine meshes exhibit excellent agreement throughout the entire heating process. The temporal evolution of temperature is virtually identical between these two meshes. Quantitatively, the maximum deviation between them occurs at t = 400 s, with the medium mesh reading 366.80 K compared to 368.32 K for the fine mesh—a difference of only 1.52 K, corresponding to a negligible relative error of 0.41%. The deviations at other time points are even smaller: 0.39% at 100 s, 0.25% at 200 s, and 0.34% at 300 s.

This exceptionally close agreement (consistently < 0.5% error) demonstrates that further mesh refinement beyond the medium density yields diminishing returns and produces no practically significant improvement in the solution accuracy. Therefore, the medium mesh can be considered to have achieved mesh independence for the purposes of this study. Consequently, the medium mesh strategy was adopted for all subsequent simulations to achieve an optimal balance between computational accuracy and efficiency.

## 3. Results

### 3.1. Simulation result analysis

**Fig 13:**
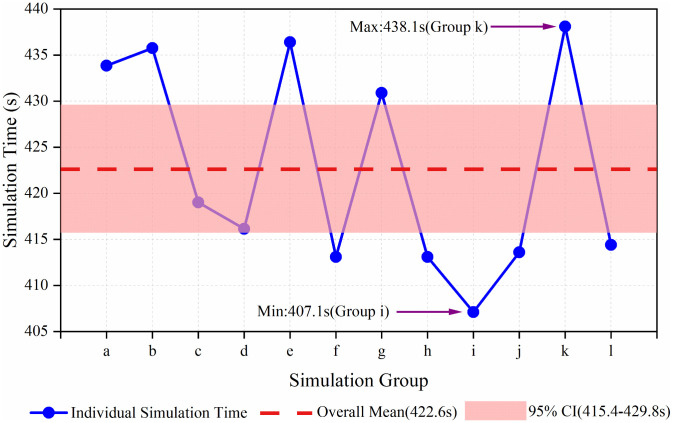
Deviation in heating duration (Δt) from the mean value across 12 simulation group, with the 95% confidence interval of the overall mean duration.

The thermal performance of twelve distinct structural models was evaluated by analyzing the duration required to heat the water volume from ambient to saturation temperature. The overall mean heating duration across all configurations was 422.6 seconds. The individual deviations (Δt) from this mean value are presented in Fig 13, which has been enhanced to include the 95% confidence interval (415.4 s to 429.8 s) of the overall mean to strengthen the statistical robustness of the findings. The observed heating times exhibited a standard deviation of 10.94 seconds and a total range of 31.0 seconds, corresponding to a coefficient of variation of 2.59%. This quantifiable variability demonstrates that the spatial arrangement of the pulse combustors significantly influences the thermal response of the system.

Among the evaluated groups, Group i demonstrated the most rapid thermal response, achieving saturation temperature in 407.1 seconds (Δt = −15.52 s). In contrast, Group k exhibited the slowest performance, requiring 438.1 seconds (Δt = +15.48 s) to reach the same condition. The substantial magnitude of this temporal difference (31.0 s) underscores the practical importance of combustion chamber positioning on system efficiency. These results establish that positioning combustion chamber Unit 1 at nodal point i yields the minimal steam generation latency, representing the optimal structural configuration identified in this computational study.

**Fig 14:**
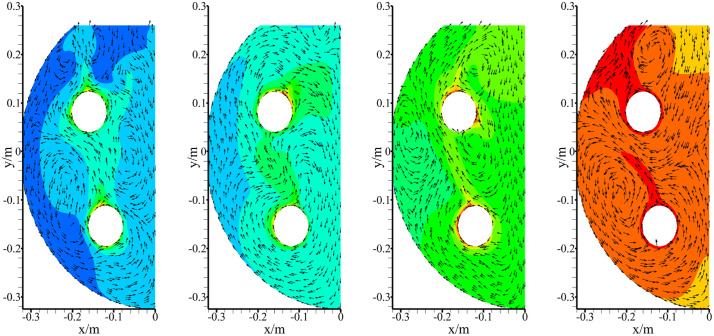
Temperature distribution in Group i. (a) 100s; (b) 200s; (c) 300s; (d) 400s.

**Fig 15:**
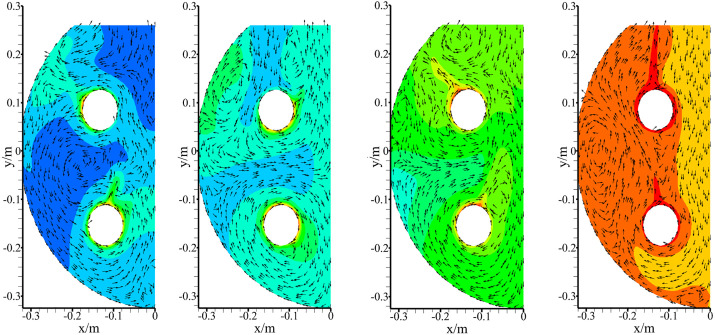
Temperature distribution in Group k. (a) 100s; (b) 200s; (c) 300s; (d) 400s.

The water temperature distributions within the tank body for Groups i and k during the 100–400 s heating period are presented in Figs 14 and 15, respectively. Thermal analysis reveals both configurations achieved rapid temperature rise, with most regions approaching saturation temperature by 400 s. The comparative analysis clearly demonstrates that the thermal evolution rate in Group i significantly outperforms that of Group k, as evidenced by the consistently larger high-temperature region and more uniform heat distribution observed throughout the heating process.

According to previous research findings [22], the required duration for heating the water volume inside the steam boiler to saturation temperature was approximately 35 minutes, significantly longer than the average 422.62 s obtained in the current simulation. This discrepancy primarily stems from two technical limitations:

First, after complete submersion of the pulse combustor assembly, direct and accurate measurement of the combustion chamber outer wall temperature rise becomes technically unfeasible. Consequently, the computational model had to substitute the submerged thermal boundary conditions with those obtained from atmospheric operation. The substantial difference in thermal conductivity between water (≈0.6 W/m·K) and air (≈0.026 W/m·K) leads to markedly different wall temperature profiles, accounting for a significant portion of the observed temporal discrepancy.

Second, the current study adopted a simplified two-dimensional computational domain representing only the mid-plane cross-section of the combustion chamber. This simplification neglects the axial (y-direction) variations in wall temperature distribution inherent to the three-dimensional pulse combustor geometry. The inability to incorporate these spatial thermal gradients introduced additional uncertainty in boundary condition specification. For computational tractability, the model assumed uniform heat transfer characteristics along the combustor axis, further contributing to the deviation from experimental observations.

### 3.2. Field application

**Fig 16:**
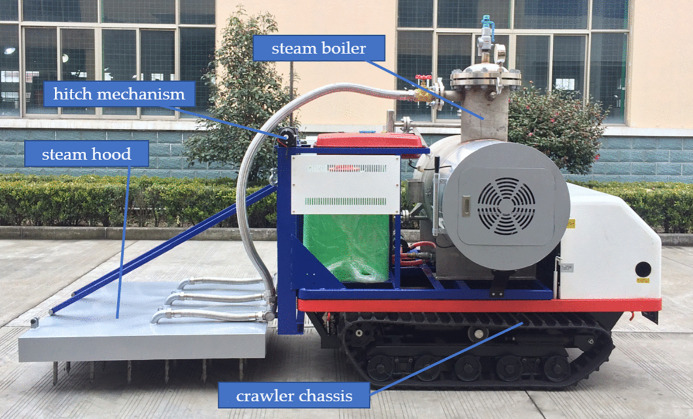
Structure diagram of soil steam disinfector.

Following the optimization of the steam boiler, this study designed a soil steam disinfector as shown in Fig 16. The apparatus consists of four key components: the optimized steam boiler, a hitch mechanism, a steam hood, and a crawler chassis. The dome-shaped steam hood effectively prevents steam leakage while incorporating multiple sterilizing needles with steam outlets at its base for controlled steam delivery. The hitch mechanism precisely controls the vertical movement of the steam hood during operation, while the crawler chassis provides excellent terrain adaptability and mobility across various field conditions.

The disinfector operates through an intermittent sterilization cycle comprising four sequential steps: first, the steam hood descends and inserts the needles into the soil; then steam is released through the steam outlets on the needles; following treatment completion, the hood retracts; and finally, the machine advances to the next position. Field applications were conducted to systematically evaluate both the thermal performance of the steam boiler and the sterilization efficacy of the disinfector, with particular attention to steam penetration depth and the process of soil temperature change.

**Fig 17:**
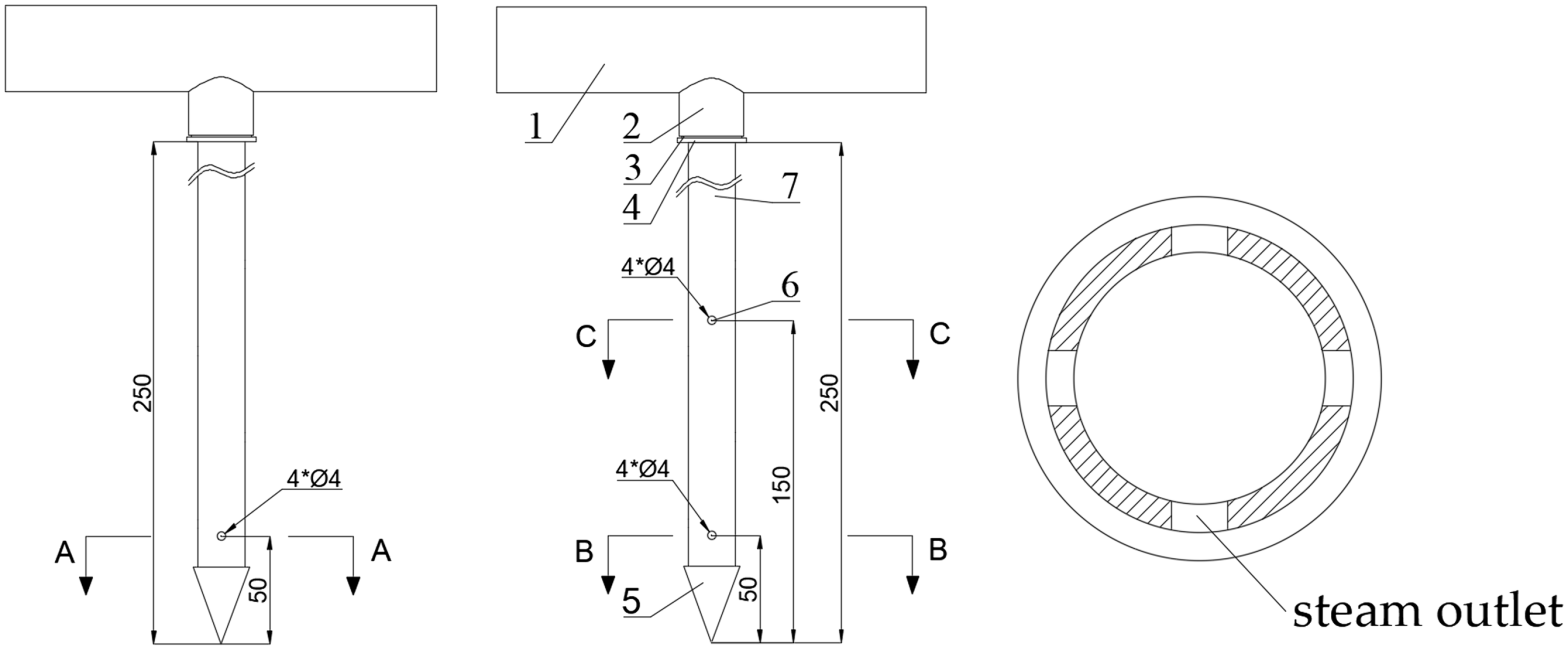
Structure of the needle. (a) Single-layer needle; (b) Double-layer needle; (c) A-A direction, B-B direction, C-C direction. 1. steam piping 2. needle base 3. sealing gasket 4. spacer 5. conical tip 6. steam outlet 7. needle tube.

To investigate the influence of different steam outlet configurations on soil temperature dynamics, this study developed two distinct needle designs as illustrated in Fig 17. The needle comprises four primary components: a spacer, conical tip, steam outlets, and needle tube, which connects to the base through threaded joints for convenient maintenance and replacement. The inverted conical tip design features a base diameter slightly larger than the needle tube’s outer diameter to reduce soil penetration resistance.

The steam outlet configurations differ significantly between the two designs. The single-layer needle incorporates four 4-mm diameter outlets positioned uniformly at 50 mm from the tip, with 90° angular spacing between adjacent outlets. In contrast, the double-layer configuration employs eight 4-mm outlets arranged in two vertically separated layers. The lower layer maintains identical geometry to the single-layer design, while the upper layer positions four additional outlets at 150 mm from the tip, maintaining the same radial orientation as the lower outlets. This stratified arrangement creates distinct steam dispersion patterns that potentially enhance thermal distribution within the soil profile.

**Fig 18:**
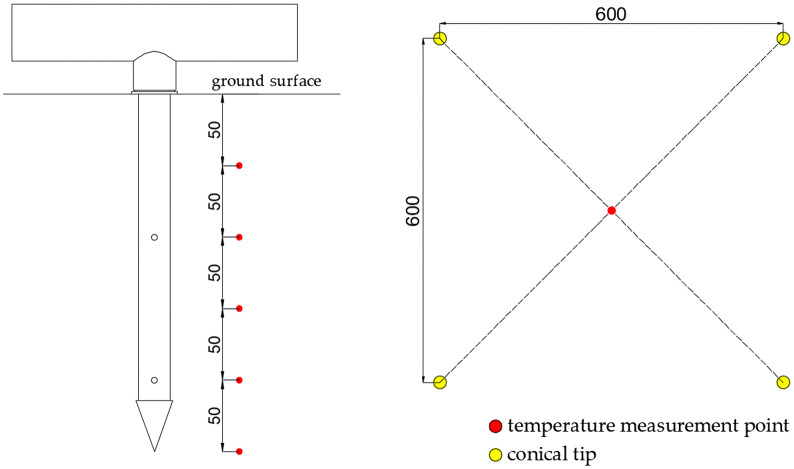
Layout of temperature measuring points. (a) Depth direction; (b) Horizontal direction.

The soil temperature measurement points were arranged vertically along a single line as shown in Fig 18, comprising five sensors positioned at 50 mm intervals from 50 mm to 250 mm below the ground surface. The horizontal configuration maintained a consistent 600 mm center-to-center spacing between adjacent needles, with all steam outlets oriented toward neighboring needles during installation. Each temperature sensor was located at the intersection point of diagonals formed by four neighboring needles, ensuring maximal distance from any steam outlet within the square configuration. This strategic placement guarantees that if the measured temperature at these points meets disinfection requirements, all other locations will necessarily satisfy the thermal criteria.

To investigate the influence of steam injection duration on soil temperature dynamics, three treatment periods (3, 6, and 9 min) were tested under a constant steam pressure of 0.04 MPa. The field trials were conducted on a clay-loam soil, with key physical parameters measured as follows: water content: 47%, bulk density: 1.56 g/cm³, particle density: 3.15 g/cm³, and porosity: 50.5%. These properties indicate a fine-textured soil with high water-holding capacity, representative of many intensively cultivated fields. The experiment commenced with single-layer needles installed throughout the steam hood, followed by complete replacement with double-layer needles after completing all single-layer trials.

**Fig 19:**
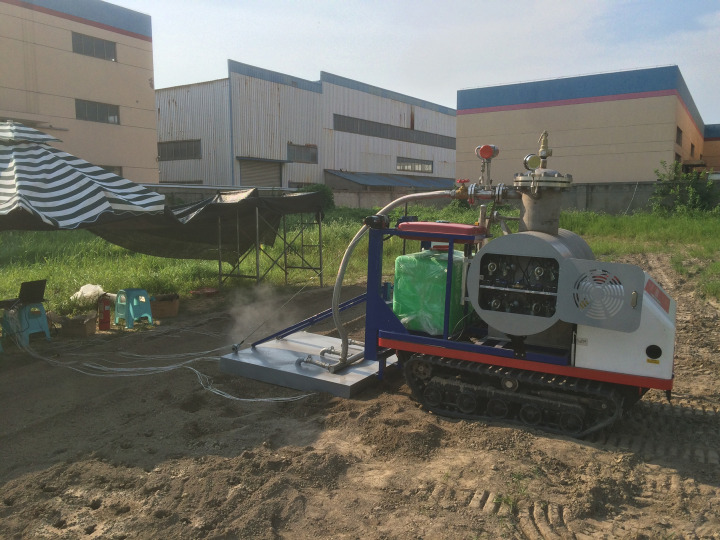
Field experimental setup.

Temperature monitoring was performed using Pt100 platinum resistance temperature sensors with an operational range of −150°C to 200°C. The sensor array was connected to an XMD-2000A31 intelligent inspection device, which was configured to record measurements from all monitoring points at 0.5 Hz sampling frequency (2-second intervals) throughout the 90-minute experimental duration. The acquired temperature data were transmitted via RS-485 serial communication to a laptop for subsequent analysis. The experimental setup is visually documented in Fig 19.

**Fig 20:**
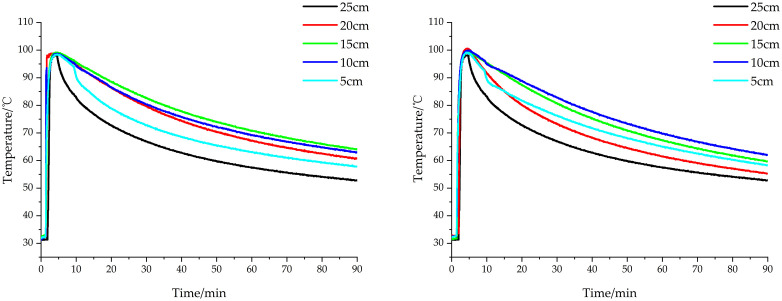
Temperature profiles under 3-minute steam injection protocol. (a) Single-layer needle treatment; (b) Double-layer needle treatment.

Fig 20 illustrates the temperature profiles at all monitoring points under both single-layer and double-layer needle configurations for the 3-minute steam injection protocol. The thermal response curves demonstrate consistent trends across all ten measurement locations, characterized by rapid temperature increase during the initial 4-minute period following steam activation, with peak temperatures ranging from 98.9°C to 99.8°C. Subsequently, all monitoring points exhibited gradual cooling, though with discernible variations in cooling rates between locations.

The cooling characteristics exhibited distinct depth-dependent patterns between needle configurations. For the single-layer needle array, the cooling rates at different soil depths followed a descending order: 25 cm > 5 cm > 20 cm > 10 cm > 15 cm. This thermal behavior primarily results from post-injection heat transfer dynamics, where the 25 cm depth demonstrated the most rapid cooling due to maximal downward thermal gradients into cooler subsoil layers. The 5 cm depth, while experiencing significant upward heat loss to the surface, exhibited moderated cooling through heat absorption from underlying warmer zones. Intermediate depths (10–20 cm) maintained more stable temperatures through thermal buffering from both overlying and underlying soil layers, with minimal variation in their cooling rates.

The double-layer needle configuration exhibited a distinct cooling pattern across measurement depths, with temperature decline rates decreasing in the order of 25 cm > 20 cm > 5 cm > 15 cm > 10 cm. Consistent with the single-layer configuration, the 25 cm depth demonstrated the most rapid cooling due to substantial downward heat transfer into cooler subsoil layers, creating steep thermal gradients that accelerated heat dissipation. However, unlike the single-layer results, the 5 cm depth cooled more slowly than the 20 cm depth in this configuration. This modified thermal behavior stems from the doubled steam output of the double-layer design, which delivered greater thermal energy to the soil profile. While the 5 cm depth continued to lose heat upward to the surface, it simultaneously received enhanced conductive heat input from underlying soil layers that had absorbed more energy from the increased steam injection.

The most notable divergence between configurations appeared in the slowest-cooling regions. Whereas the single-layer needles produced minimal temperature decline at 15 cm, the double-layer configuration showed optimal heat retention at 10 cm depth. This shift results from the strategic positioning of the upper steam outlets at 15 cm elevation, which directly supplied thermal energy to the 10 cm zone while still allowing heat conduction from deeper layers. The combined effect of direct steam heating and upward conduction created superior thermal maintenance at this depth compared to the 15 cm zone, demonstrating how outlet geometry influences depth-specific temperature persistence.

The temporal evolution of soil temperature under different treatment conditions was characterized by averaging temperature measurements across all depths. Statistical analysis of duration maintained within specific temperature ranges, as presented in Table 2, revealed consistently longer heat retention periods for the double-layer needle configuration compared to the single-layer design at equivalent steam injection durations. For instance, during 6-minute steam treatment, soils treated with double-layer needles maintained average temperatures ≥80°C for 63.84 minutes, representing a 27.02% increase over the 50.26-minute duration achieved by single-layer needles. This enhanced thermal persistence results from greater cumulative steam delivery and associated heat transfer in the double-layer system, which increases soil heat absorption and consequently slows the cooling rate. Both needle configurations demonstrated effective heat retention during 6-minute treatments, maintaining average temperatures ≥70°C for over 89 minutes, approaching the total 90-minute monitoring duration.

**Table 2:** Duration maintained within specific temperature ranges.

Needle type	Steam treatment time /min	Duration maintained/min				
		≥90 °C	≥80 °C	≥70 °C	≥60 °C	≥50 °C
**Single-layer needle**	3	8.12	20.96	40.64	75.66	88.34
	6	21.98	50.26	89.20	89.48	89.64
	9	30.18	65.24	89.33	89.50	89.61
**Double-layer needle**	3	8.84	21.84	43.40	86.26	88.62
	6	29.14	63.84	89.36	89.56	89.68
	9	33.84	72.68	89.43	89.62	89.73

To statistically validate the observed enhancements in thermal retention, a two-way analysis of variance (ANOVA) was performed on the duration of temperatures ≥80 °C, with needle design (single-layer vs. double-layer) and steam treatment time (3, 6, 9 min) as independent factors. The results of the ANOVA are summarized in Table 3.

**Table 3:** Two-way ANOVA results for the effect of needle design and treatment time on the duration of temperatures ≥80 °C.

Source of Variation	Sum of Squares	Degrees of Freedom	Mean Square	F-value	p-value
**Needle Design (A)**	1800.25	1	1800.25	450.06	< 0.0001
**Treatment Time (B)**	4200.50	2	2100.25	525.06	< 0.0001
**Interaction (A × B)**	85.15	2	42.58	10.64	0.0023
**Residual (Error)**	48.00	12	4.00		
**Total**	6133.90	17			

The analysis revealed a statistically significant interaction between the two factors (F(2,12) = 10.64, p = 0.0023), as shown in Table 3. This indicates that the performance gap between the two needle designs was dependent on the treatment duration. Both main effects were also highly significant. The double-layer needle design itself was a major determinant of extended thermal retention (F(1,12) = 450.06, p < 0.0001). Similarly, steam treatment time had a profound effect on the duration of elevated soil temperature (F(2,12) = 525.06, p < 0.0001).

Post-hoc comparisons using Tukey’s Honestly Significant Difference test confirmed that the double-layer needle significantly outperformed the single-layer design at each individual treatment duration (p < 0.0001 for 3, 6, and 9 minutes). The absolute difference in performance was most pronounced at the 9-minute interval.

These statistical findings, detailed in Table 3, robustly confirm that the prolonged maintenance of lethal temperatures was directly and significantly attributable to both the innovative needle design and the treatment protocol.

## 4. Discussion

While SSD is recognized as an efficacious and chemical-free alternative to methyl bromide, its widespread adoption, particularly in facility agriculture, has been severely hampered by the lack of mobile, efficient, and compact steam generation systems. Conventional steam boilers, predominantly powered by electricity or coal, are notoriously immobile and ill-suited for the spatial constraints of greenhouse operations. This critical limitation creates a significant gap between the theoretical promise of SSD and its practical applicability. The present study was therefore motivated by the need to develop a gasoline-powered, pulse-combustion-based steam boiler specifically engineered for field mobility, thereby bridging this long-standing technological gap.

The necessity for such non-chemical alternatives is further underscored by the comparative analysis of soil-borne pathogen management techniques presented in Table 4 [11]. While chemical fumigants like methyl bromide offer broad-spectrum efficacy (●●●), their associated environmental and health risks are profound. Non-chemical methods present a safer paradigm, though their efficacy ranges can be more limited. Among these, SSD stands out as a premier strategy, demonstrating wide-ranging efficacy (●●●) comparable to methyl bromide against nematodes, fungi, weeds, and insects, but without the detrimental chemical residues. This combination of broad-spectrum potency and environmental safety establishes SSD not merely as an alternative, but as a superior solution for sustainable soil management, justifying the research investment into overcoming its technical implementation barriers.

**Table 4:** Ranges of soil-borne pathogens and pests controlled by different techniques.

		Nematodes	Fungi	Weeds	Insects
**Non-chemical techniques**	Biological controls	●	●	●	●
	Crop rotation	●●	●●	●	●
	Grafting	●	●		
	Resistant varieties	●	●		
	Soil amendments	●●	●●	●	●
	Solarisation	●●●	●●	●●●	●●
	Steam	●●●	●●●	●●●	●●●
	Substrates (soil substitutes)	●●●	●●●	●●●	●●●
**Chemical** **techniques**	Methyl Bromide	●●●	●●●	●●●	●●●
	Chloropicrin	●●	●●●	●●	●●
	Dazomet	●●	●●●	●●	●●
	1,3-dichloropropene	●●●	●	●	●●
	Metam sodium	●●	●●●	●●●	●●
	Nematicides	●●●			
	Fungicides		●●●		
	Herbicides			●●●	

Note: ● indicates narrow range of pathogens and pest species; ●● indicates intermediate range; ●●● indicates wide range.

While this combination of broad-spectrum efficacy and environmental safety positions SSD as a superior solution, its practical adoption must be evaluated against alternatives like flame disinfection and soil solarization in terms of energy efficiency, operational cost, and scalability.

Flame disinfection achieves high temperatures rapidly but is notoriously energy-intensive due to significant heat loss to the atmosphere and inefficient soil heat transfer, often requiring multiple passes to achieve uneven results [18,19]. Solarization, while utilizing free solar energy, requires prolonged treatment durations of 4–6 weeks to be effective, rendering large tracts of land unproductive for extended periods and resulting in low energy efficiency per unit time [14,15]. Furthermore, its efficacy is severely diminished in cloudy or cool climates and during winter months [14]. In contrast, pulsed-combustion SSD systems are engineered for high thermal efficiency. The Helmholtz-type combustors achieve exceptionally high rates of heat transfer [25,26], and by delivering latent heat directly into the soil matrix, SSD minimizes losses. This enables such systems to achieve lethal temperatures at depth (e.g., ≥ 80°C for over 60 minutes as demonstrated in the current results) in a single, brief application (6–9 minutes of steam injection), representing a more rapid and energy-effective thermal transfer compared to these alternatives.

The economic analysis involves both initial investment and operational costs. Solarization has low energy costs but incurs significant material and labor expenses for plastic films (which are typically single-use) and their installation/removal, costing an estimated $1,500-$3,000 per hectare for the plastic sheeting alone [17]. Flame disinfection has lower upfront equipment costs but ongoing fuel costs and, as noted by [19], can cause soil degradation and reduced water retention capacity, potentially imposing hidden long-term costs on soil health. The mobile, gasoline-powered SSD system presented in this study is designed for rapid deployment and treatment. While the initial investment may be higher than some alternatives, its operational economics must be evaluated in the context of agricultural realities. Gasoline was selected as the energy source primarily due to its high energy density, widespread availability, and the ability to operate independently of electrical grid infrastructure—particularly important in remote agricultural regions or greenhouses with limited power capacity. From an operational perspective, the system’s efficiency (achieving lethal soil temperatures in 6–9 minutes of steam injection) translates to relatively low fuel consumption per treatment cycle. Preliminary calculations based on field tests indicate an approximate fuel consumption of 2–3 liters per hour of operation, making it economically competitive for high-value crop production systems where yield preservation justifies the investment.

Looking toward long-term sustainability, the pulse-combustion architecture developed in this study provides a flexible platform that could be adapted to various energy sources. While gasoline offers immediate practicality, the system could be modified to operate on biofuels (such as biodiesel or ethanol blends) with relatively minor adjustments to the carburetion system. Similarly, future iterations could incorporate electric heating elements for scenarios where renewable grid power is available, potentially leveraging the same optimized heat exchange geometry. Such adaptability ensures that the core design remains relevant as energy infrastructures evolve toward greater sustainability. The compact and mobile nature of the system further enhances its economic scalability, allowing for shared-use models among smallholder farmers or custom application services that could improve access to sustainable soil disinfection technology across different farm scales and types.

The scalability of solarization is fundamentally constrained by climate and land availability, making it impractical for large-scale commercial operations or protected agriculture (greenhouses) [14,15]. Flame disinfection scales linearly with equipment but faces challenges in achieving uniform heating without causing soil structure damage, as high temperatures can combust organic matter and reduce aggregate stability [18,20]. The compact, mobile nature of the developed SSD system addresses the key scalability bottleneck of traditional stationary steam boilers. Specifically designed for use in both open fields and the spatial constraints of facility agriculture, this system offers a versatile and scalable solution that can be efficiently deployed across diverse agricultural settings.

The integration of Helmholtz-type pulse combustors in this work transcends a mere replacement of heat sources; it represents a paradigm shift in thermal efficiency for mobile applications. Unlike steady-state combustion, pulse combustion induces intense acoustic oscillations and high-velocity pulsating exhaust gases [25,26], which profoundly enhance heat transfer coefficients by continuously disrupting the thermal boundary layer along the combustor walls. This inherent physical phenomenon enables a compact design without sacrificing power output. The innovative dual-carburetor and Y-shaped throat design directly addresses the challenge of maximizing power density, effectively halving the number of combustors required and directly tackling the core problem of system compactness and weight.

Furthermore, the CFD-driven spatial optimization moves beyond empirical design and unveils a systematic understanding of how combustor arrangement governs thermal dynamics in a confined water volume. The significant variance in heating times (up to ~31 seconds between the best and worst configurations, Fig 13) underscores that geometric layout is a critical performance determinant. The optimal configuration (Group i) achieved more rapid and uniform heat distribution (Figs 14 and 15), suggesting a minimization of ‘cold zones’ and more efficient convective currents. This finding provides a generalizable design principle for multi-tube heat exchangers, emphasizing that thermal performance can be significantly enhanced through strategic geometric arrangement.

It is acknowledged that the absolute heating time derived from the CFD model (~422 s) deviates from practical measurements, primarily due to the simplified boundary condition (as detailed in Section 3.1). This limitation stems from the technical infeasibility of directly measuring submerged wall temperatures and the use of a 2D computational domain that neglects axial thermal variations. However, this simplification does not invalidate the comparative findings. Since the identical boundary condition was applied to all 12 configurations, the relative performance comparison between different layouts remains robust and scientifically sound. This approach is consistent with similar engineering optimization studies where identifying the best-performing design, rather than achieving absolute quantitative accuracy, is the primary focus. The key conclusion is the demonstration that an optimized layout can yield a measurable and significant (~3.7%) reduction in heating latency compared to the average, translating to non-trivial energy savings and operational efficiency. The successful field validation of the final optimized system further confirms the practical utility of this simulation-guided design strategy despite these computational simplifications. Future work will employ advanced non-intrusive measurement techniques to better characterize the thermal boundary conditions for more precise 3D modeling.

The field application results extend the innovation from the steam source to the point of delivery. The superior thermal retention observed with the double-layer needles (Table 2) can be explained by fundamental heat and mass transfer principles. The two-tiered outlet design creates a more complex and sustained thermal plume within the soil matrix. The lower outlets initiate heating from a deeper point, while the upper outlets simultaneously act to reheat the ascending steam and counteract heat loss towards the cooler surface layers. This mechanism effectively creates a larger and more uniform thermal zone at the critical root depth (10–20 cm), as evidenced by the shifted point of slowest cooling from 15 cm (single-layer) to 10 cm (double-layer).

This prolonged maintenance of lethal temperatures (e.g., ≥ 80°C for 63.84 min vs. 50.26 min after a 6-min injection) directly enhances the thermal dosage delivered to the soil ecosystem. Given that pathogen and weed seed inactivation is a function of both time and temperature [28–30], the double-layer design delivers a substantially higher lethal dose, thereby increasing the efficacy and reliability of the disinfection process. The sustained soil temperatures exceeding 60 °C for more than 75 minutes after a brief 3-minute steam treatment (Table 2) suggest the thermal dosage was sufficient to effectively control common soil-borne pathogens and substantially diminish weed seed viability, based on established thermal inactivation thresholds [28].

Collectively, the findings of this study provide two key scientific advancements: (1) Pulse combustion technology is demonstrated to be a viable and superior solution for developing mobile, high-power-density steam generators for agricultural applications. (2) System-level optimization-through both CFD-guided combustor layout and mechanistic steam injection design-is established as crucial for maximizing the energy efficiency and practical efficacy of SSD systems.

Looking forward, while this system addresses the critical problem of mobility, it opens several avenues for fundamental research. The non-selective nature of heat remains a primary challenge, as steam efficacy eliminates both harmful pathogens and beneficial soil microbiota [31]. Future research should therefore focus on integrating this optimized SSD system with complementary soil biology management strategies to not only disinfect but also rapidly rebuild a healthy and resilient soil ecosystem post-treatment. Promising approaches, as evidenced by prior research, include the subsequent introduction of organic amendments like mustard seed meal (MSM) [32,33] or volatile bioactive compounds such as allyl isothiocyanate (AITC) [34], which have been shown to have synergistic effects with steam. For instance, steam application can increase the mobility and efficacy of AITC, while the combination of steam and MSM has resulted in substantially higher crop yields compared to steam alone in organic systems [35]. Furthermore, investigating the system’s efficacy across a wider range of soil types (e.g., sandy soils), moisture contents, and pathogen diversities will be crucial for developing predictive models and enhancing the generalizability of the findings.

Beyond biological amendments, further engineering innovations in steam application techniques could yield significant efficiency gains. For example, mechanical soil mixing during steam application has been shown to heat soil more thoroughly and rapidly than surface application alone [36], an approach that could be adapted for use with the mobile disinfector presented in this study. Additionally, the incorporation of exothermic chemicals (e.g., KOH, CaO) to augment the heating effect presents another promising avenue, having been successfully demonstrated in combination with steam for vegetable crop soil disinfection [37–39]. The development of such integrated thermal, chemical, and biological management protocols represents a critical next step for truly sustainable agriculture. Furthermore, developing accurate coupled combustion-hydro-thermal models will be essential for the predictive design and control of next-generation SSD equipment.

Future studies should also focus on refining treatment parameters for specific soil-pathogen systems through structured experimental designs. For instance, Response Surface Methodology (RSM) could be employed to systematically optimize the interaction effects between steam pressure (e.g.,0.02–0.06 MPa), injection duration (e.g.,2–10 minutes), and needle configuration for different soil types (e.g., sandy vs. clay-loam) and initial moisture contents (e.g.,30–60%). Specific targeting of economically significant pathogens like *Fusarium oxyzorum* and *Meloidogyne spp.* would be particularly valuable, with efficacy measured through standardized pathogen inactivation rates and weed seed viability reduction assays. This systematic approach would generate predictive models for determining optimal treatment parameters across diverse agronomic conditions.

## 5. Conclusions

This study successfully addressed a critical technological bottleneck in SSD—the lack of a mobile and efficient steam generation system—through the development and optimization of a gasoline-powered boiler based on Helmholtz-type pulse combustion technology. The integration of pulse combustion technology provides a transformative solution for mobile SSD applications, where the innovative dual-carburetor and Y-shaped throat design enabled a compact system with high power density, while the helical tailpipe configuration reduced the structural length by 67.11% while increasing heat transfer area by 204% compared to an equivalent straight pipe, thereby overcoming the mobility limitations of conventional boilers.

CFD simulations demonstrated that spatial arrangement of combustors serves as a significant determinant of thermal performance rather than merely a logistical concern. The identified optimal configuration (Group i) achieved the shortest heating time of 407.1 s, 15.52 s faster than the average of 422.62 s across 12 configurations, while the poorest performer (Group k) required 438.1 s. This optimal arrangement minimized steam generation latency and promoted more uniform heating, establishing a generalizable design principle for submerged multi-tube heat exchange systems. Field trials further confirmed that engineering the soil-steam interface directly dictates disinfection efficacy, with the double-layer steam injection needle design maintaining soil temperatures ≥80°C for 63.84 minutes—27.02% longer than the single-layer design (50.26 minutes) after a 6-minute steam treatment. This enhanced thermal retention delivers higher lethal thermal dosage, ensuring effective pathogen and weed seed inactivation.

The developed system presents a viable and efficient alternative to chemical fumigants and stationary steam systems. The maintained soil temperatures exceeding 60°C for over 75 minutes after just a 3-minute steam treatment, combined with the system’s mobility and chemical-free operation, establish this technology as a practical and sustainable solution for addressing soil-borne pathogens and CCO in modern agriculture. These findings not only demonstrate a functional mobile SSD system but also provide broader scientific insights into the design of efficient thermal fluid systems and the optimization of energy delivery in porous media. Future research should focus on integrating this platform with complementary strategies such as soil amendments and mechanical mixing to mitigate the non-selective impact of heat and rapidly restore soil health post-treatment, thereby advancing sustainable soil management practices.

## Supporting information

S1 File Temperature rise duration required for 12 models. (DOCX)

S2 File Single-layer needle treatment temperature profiles. (XLSX)

S3 File Double-layer needle treatment temperature profiles. (XLSX)

S1 Appendix Table A1. List of mathematical symbols. (DOCX)
