# Supplementary material for: Optimized steam boiler for soil steam disinfection: Structural design, CFD simulation, and field application
Source: PLoS One. 2025 Dec 26;20(12):e0340080. doi: 10.1371/journal.pone.0340080 (PMC12742748; doi:10.1371/journal.pone.0340080)
Supplement: S1 File — (DOCX) [file pone.0340080.s001.docx]

Optimized Steam Boiler for Soil Steam Disinfection: Structural Design, CFD Simulation, and Field Application

Sipu Pan

**S1 File.** **Temperature rise duration required for 12 models.**

| Group | Time/s |
| --- | --- |
| a | 433.85 |
| b | 435.75 |
| c | 419 |
| d | 416.15 |
| e | 436.4 |
| f | 413.1 |
| g | 430.9 |
| h | 413.1 |
| i | 407.1 |
| j | 413.6 |
| k | 438.1 |
| l | 414.4 |
