# Supplementary material for: Optimized steam boiler for soil steam disinfection: Structural design, CFD simulation, and field application
Source: PLoS One. 2025 Dec 26;20(12):e0340080. doi: 10.1371/journal.pone.0340080 (PMC12742748; doi:10.1371/journal.pone.0340080)
Supplement: S1 Appendix — (DOCX) [file pone.0340080.s004.docx]

Appendix A

Table A1. List of mathematical symbols.

| Symbol | Definition Description | Unit |
| --- | --- | --- |
| *q_r_* | volumetric heat release rate of the combustion chamber | kW/m^3^ |
| *P_r_* | power output of the pulse combustor | kW |
| *V_r_* | combustion chamber volume | m^3^ |
| *D_r_* | internal diameter of the combustion chamber | m |
| *L_r_* | length of the combustion chamber | m |
| *S_w(i)_* | cross-sectional area of the i^th^ tailpipe | m^2^ |
| *S_r_* | combustion chamber cross-sectional area | m^2^ |
| *D_w_* | individual tailpipe inner diameter | m |
| *θ* | coefficient |  |
| *a_l_* | liquid phase volume fraction in the fluid | % |
| *a_g_* | vapor phase volume fraction in the fluid | % |
| $\boldsymbol{\rho}_{\boldsymbol{m}}$ | density of the two-phase mixture | kg/m³ |
| *t’* | time | s |
| ${\vec{\boldsymbol{v}}}_{\boldsymbol{m}}$ | velocity vector of the two-phase flow | m/s |
| *p* | pressure | Pa |
| $\vec{\text{g}}$ | gravitational acceleration vector | m/s² |
| $\vec{\text{F}}$ | body force vector | N/kg |
| $\boldsymbol{\mu}_{\boldsymbol{m}}$ | dynamic viscosity of the two-phase flow | Pa·s |
| $\text{φ}_{\text{k}}$ | volume fraction of phase k | % |
| $\boldsymbol{\rho}_{\text{k}}$ | density of phase k | kg/m³ |
| ${\vec{\text{v}}}_{\text{dr,k}}$ | drift velocity vector of phase k | m/s |
| *h_k_* | specific enthalpy of phase k | J/kg |
| *v_k_* | velocity of phase k | m/s |
| $\text{λ}_{\text{eff}}$ | effective thermal conductivity | W/(m·K) |
| *T’* | temperature | K |
| S_E_ | source term | J/kg |
| *M_l_* | liquid phase mass source term | kg/(m³·s) |
| *ω* | relaxation factor | s⁻¹ |
| $\text{ρ}_{\text{l}}$ | liquid phase density | kg/m³ |
| *M_g_* | vapor phase mass source term | kg/(m³·s) |
